# Evaluation of Survival Rates of Dental Implants and the Risk Factors: A Retrospective Follow-Up Study

**DOI:** 10.7759/cureus.55360

**Published:** 2024-03-01

**Authors:** Shailesh Jain, Addugala Hemavardhini, Maitreyi Ranjan, Neeta Pasricha, Sahil S Thakar, Keyur J Soni, Sahba Hassan, Keshav Goyal, Diksha Singh

**Affiliations:** 1 Department of Prosthodontics and Crown and Bridge, Sharda University, Greater Noida, IND; 2 Department of Prosthodontics and Crown and Bridge, G.Pulla Reddy Dental College & Hospital, Kurnool, IND; 3 Department of Microbiology, School of Dental Sciences, Sharda University, Greater Noida, IND; 4 Department of Prosthodontics and Oral Implantology, Institute of Technology & Science (I.T.S) Dental College, Muradnagar, IND; 5 Department of Public Health Dentistry, Himachal Dental College, Mandi, IND; 6 Department of Orthodontics and Dentofacial Orthopedics, Ahmedabad Dental College & Hospital, Ahmedabad, IND; 7 Department of Prosthodontics and Crown and Bridge, DJ College of Dental Sciences & Research, Ghaziabad, IND; 8 Department of Prosthodontics and Crown and Bridge, Shree Bankey Bihari Dental College, Ghaziabad, IND; 9 Department of Periodontics and Implantology, Shree Bankey Bihari Dental College, Ghaziabad, IND

**Keywords:** implantology, dentistry, risk factors, survival rate, implant placement

## Abstract

Introduction

The current research sets out to assess implant early survival rates and identify relevant parameters.

Methods

The research spanned the years 2021 and 2022 and included all individuals who had dental implants. Various criteria, such as age, sex, maxilla/mandible, implant location, immediate implant, implant diameter, implant length, and others, were used to determine the implant survival rate in the research. A multiple logistic regression model was used to show the risk variables for early survival rates of implants, and components with p < 0.05 were further included after the Chi-square test was employed to filter them.

Results

The current research included 128 patients who had a single implant procedure, including 70 males and 58 females. The early survival rate was 91.40%, and 117 implants were retained after implantation. Risk variables that were shown to be associated with early survival rates were patients aged 30-60 years (OR: 2.542), immediate implant placement (OR: 3.742), and implant length less than 10 mm (OR: 3.972).

Conclusions

Age, tooth location, implant length, and immediate implantation were risk variables that contributed to our subjects’ above 91% early survival rate of implants.

## Introduction

Implants are considered the “third set of teeth” because of their resemblance to natural teeth in appearance, comfort, and chewing efficiency [1]. One of the most common ways to restore lost teeth is through dental implant treatment. Although the long-term success of dental implants has been reported in many studies, several risk factors associated with implants, surgery, and patient-related components may disturb long-term implant survival [2]. The current tendency is to improve aesthetics and patient comfort, thanks to the fast progress of dental implant therapies. It is very beneficial to establish healthy papillae and gingival contours around implants. This is particularly true for individuals who expose soft tissue when they talk or smile [3,4].

Implant-supported prostheses have a high survival rate, which has been proven in many studies, but they also have shown certain complications, and their longevity is in question as they have biological complications as well as complications in prosthetic maintenance and restorative issues [5,6]. While evaluating whether or not to use a procedure like dental implant placement, it is necessary to review the literature and analyze the data. Literature helps clinicians assess the clinical course of the treatment, as well as in decision-making and diagnosis, to plan the treatment procedure and protocol [7,8].

One- or two-year follow-up clinical studies have shown that implant failure mainly occurs during the early stages of implant placement, like osseointegration and mastication. Most studies conducted on survival and success rates are concentrated toward one or two years after immunization [9,10]. Many studies reported the possible risk factors for implant failure, like systemic diseases, bone level, adverse habits, implant pattern, surgical technique, early loading, etc. [11,12]. Implant early survival rates are affected by a number of circumstances; hence, it is essential to examine and analyze these characteristics. By looking back at data from 2018-2020, we were able to calculate the early survival rates of implants and identify the variables that had an impact on them.

## Materials and methods

Participants in this research were those who had dental implant surgery for a single tooth between April 2021 and March 2022. The procedure lasted for a year. Approval from the school’s ethics board was necessary for this project. The research study was approved by the Institutional Ethics Committee of Sharda University (approval number IEC/SU/2021/22).

The study inclusion criteria included patients who were eligible for a single-tooth dental implant, had no medical conditions that would prevent them from having the procedure done, and gave their informed consent.

However, exclusion criteria include patients who were not eligible for the study due to certain conditions. These conditions included hepatobiliary disease, uncontrolled diabetes mellitus, severe periodontitis, alcoholism, substance abuse, and not following the doctor’s instructions during osseointegration after implantation. It was advised that patients refrain from smoking after implant placement, particularly while the incision was healing. Additionally, patients were not included in the research if they failed to attend their scheduled review sessions or answer any questions sent following their implantation.

**Figure 1:**
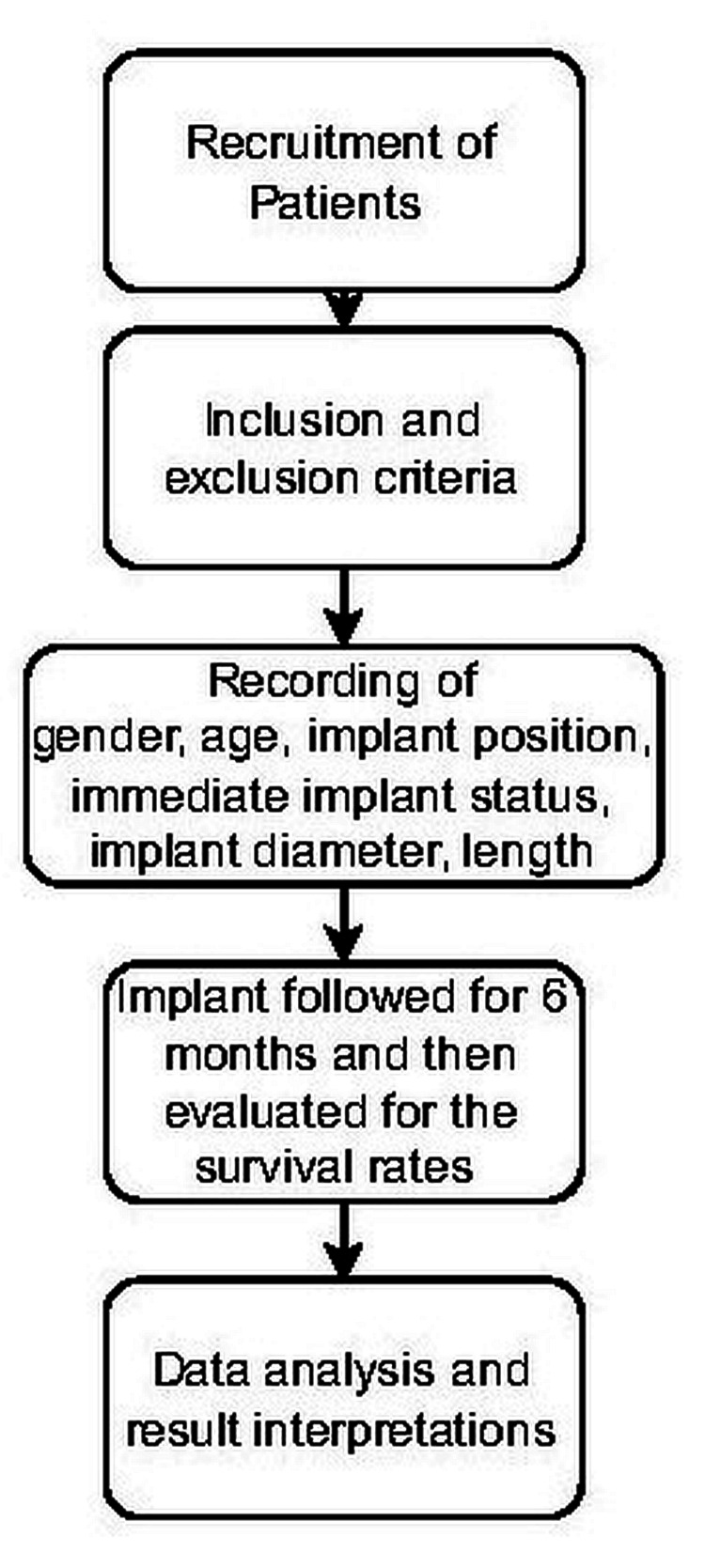
Flowchart of the methodology

During the initial step of implant surgery, the dentists recorded the gender, age, implant position, immediate implant status, implant diameter, length, and overall condition in the patient’s personal implant file. All prostheses were repaired during the second stage of surgery, which was performed by the dentist three to six months after the implants were placed (Figure 1).

We used IBM SPSS Statistics for Windows, Version 22.0 (Released 2013; IBM Corp., Armonk, NY, USA) for statistical analysis after recording baseline attributes and entering data into a Microsoft Excel spreadsheet (Microsoft Corporation, Redmond, WA, USA). The researchers analyzed the data and presented descriptive statistics in terms of frequency and percentages for gender, age, maxilla/mandible, implant location, instantaneous implant, implant diameter, and other associated parameters. A Chi-square test was used to ascertain the distinctions between and among the various groups. To further determine whether there was a simultaneous influence on failure rate, the variables with significant differences (p < 0.05) in Chi-square tests were included in the multivariate logistic regression model.

## Results

This research comprised 128 patients with single-tooth implants: 70 were male, and 58 were female. The patients had an average age of 42.00 ± 5.4 years. There were 117 retained implants after implant insertion, with a survival rate of 91.60%. Infection (5), periodontal disease (2), inadequate oral hygiene (2), and incorrect occlusion owing to early stress (2) were the main reasons for failure in the 11 implants that were evaluated (2). Research was conducted to identify potential factors that impact the longevity of dental implants. The survival rate for males was 90.00%, while the survival rate for females was 93.11%, according to the data in Table 1.

**Table 1:** Comparison of patients’ basic characteristics with survival rate

Variables		Total			Survival rate		p-Value
		Frequency	Percentage		Frequency	Percentage	
Gender	Male	70		54.68	63	90.00	0.051
	Female	58		45.31	54	93.11	
Age	<40 years	54		42.18	51	94.55	0.043
	>40 years	74		57.81	66	89.19	
Dentition	Maxilla	68		53.12	61	89.70	0.065
	Mandible	60		46.87	56	93.33	
Implant position	Anterior	32		28.00	28	87.50	0.001
	Premolar	19		14.84	16	84.21	
	Posterior	77		60.15	73	94.80	

The effectiveness of the implant was also affected by its placement. Premolars had an 84.21% survival rate, molars an 87.50% rate, and front teeth an 84.21% rate. The rates of survival varied considerably across the various implant lengths. In terms of implant length, the survival rates were 81.81% for implants longer than 13 mm, 92.59% for those between 10 and 13 mm, and 92.75% for those less than 10 mm. The survival rates of patients who had their implants placed immediately were much lower than those of patients whose implants were placed later (Table 2).

**Table 2:** Implant factors that affected the early survival rate

Variables		Total			Survival rate		p-Value
		Frequency	Percentage		Frequency	Percentage	
Immediate implant	Yes	24		18.75	17	77.27	0.001
	No	104		81.25	99	95.19	
Implant length	<10 mm	69		53.9	64	92.75	0.003
	10-13 mm	37		28.9	35	94.59	
	>13 mm	22		17.18	18	81.81	
Implant diameter	<4.2 mm	68		53.12	60	88.23	0.056
	>4.2 mm	60		46.87	57	95.00	

We used multivariate logistic regression to examine variations in early survival rates by incorporating patient age, implant location, instantaneous implantation status, and implant length, among other variables. The survival rates of individuals under the age of 30 differed significantly from those of patients older than 40. This outcome was statistically significant for the immediate implant. The survival rates were not significantly affected by other factors, such as implant length or location (premolar, molar, or anterior teeth; Table 3).

**Table 3:** Multivariable logistic regression analysis for survival rates

Variables		OR	95% CI	p-Value
Age	<40 years	2.542	1.243-3.753	0.029
	>40 years	Reference	Reference	
Implant position	Anterior	Reference	Reference	0.754
	Premolar	0.835	0.537-1.487	
	Posterior	0.745	0.385-1.742	
Immediate implant	Yes	Reference	Reference	0.002
	No	0.742	0.436-0.876	
Implant length	<10 mm	3.972	1.482-5.935	0.026
	10-13 mm	1.213	0.845-1.542	
	>13 mm	Reference	Reference	

## Discussion

In patients with complete and partial edentulism, implant therapy is considered a safe and reliable method. Since the average life expectancy is progressively increasing, patients need more dental implants. Long-term success and survival rates of osseointegrated implants have been reported in several studies [13]. The success and success rates of dental implants placed by specialists have been well documented in numerous studies. The use of dental implants as a replacement for missing teeth has been increasing steadily, probably owing to their high predictability and survival rates, as reported in numerous studies [14,15].

Overall, the survival rates of the implants in the long-term evaluation presented here are within the reported rates in the literature, both at the implant level and at the patient level. It is important to emphasize, though, that proper analysis with cumulative survival analysis is of utmost importance when reporting on long-term results for such large cohorts [16]. According to the present study, the reason for early implant failure is patients’ inability to maintain oral hygiene due to their work schedule, which is also reported in clinical literature [14]. Immediate implant placement is more susceptible to microorganisms, which leads to poor healing and a higher risk of implant failure; therefore, primary stability and proper sealing of hard and soft tissues are required [17]. A study by Meijer et al. [18] on immediate implantation in the molar area has shown a survival rate of 73.3%. Another study found that a higher survival rate is seen with delayed implant placement [19]. Improving knowledge, training, diagnostic ability (the ability to evaluate implant difficulty and make a proper treatment plan), proper case selection, and surgical skills are key features in increasing the success rate as well as reducing early implant failure. This will help us find solutions and methods to increase early implant success in clinical practice and provide some references for prospects. Early signs of implant failure can be identified by long-term evaluation and follow-up for each patient, which will help in early detection and prompt treatment, leading to beneficial treatment outcomes [20].

To identify the barriers and present implementation strategies to enhance the uptake of these approaches, a comprehensive analysis of current practices and their feedback was conducted. It is about trying to implement the knowledge we have into our daily practice, ensuring our patients are receiving evidence-based treatments. The employment of evidence-based research is required to provide optimal treatment for patients. Research is undeniably critical for patient care; however, we must be able to apply it, and therefore, there is a need for more implementation science in dentistry.

The study has some limitations: it included 128 patients who underwent single implant procedures. While this sample size is adequate for initial observations, it may not fully represent the diverse patient population encountered in clinical practice. Additionally, the distribution of age, gender, and other demographics among the participants could affect the generalizability of the results to broader populations. Longer-term evaluations are necessary to ascertain the durability and success of these implants over extended periods. This study employed a retrospective design, which may introduce inherent biases and limitations associated with data collection from patient records. Prospective studies with standardized protocols can provide more robust and controlled data. The inclusion of only patients who had a single implant procedure could introduce selection bias. Patients who require multiple implants may have different risk profiles or treatment outcomes, and their exclusion limits the study’s applicability to broader clinical scenarios. However, researchers and clinicians should be mindful of these limitations when interpreting the results and consider conducting further investigations to enhance the comprehensiveness and applicability of findings in clinical practice.

## Conclusions

This study reported on an analysis of the success rates, risk factors, and survival rates of dental implants in patients who had a single implant placement. The early survival rate of implants in our cohort was on the higher side. Although the results are promising and encouraging in terms of survival, it is important to emphasize the potential risk factors and consider them before dental implant placement.
